# The Screening of Critical Related Genes in Celiac Disease Based on Intraepithelial Lymphocytes Investigation: A Bioinformatics Analysis

**DOI:** 10.31661/gmj.v8i0.1407

**Published:** 2019-08-14

**Authors:** Mohammad Rostami-Nejad, Reza vafaee, Mohammad Javad Ehsani-Ardakani, Nika Aghamohammadi, Aliasghar Keramatinia, Saeed Abdi, Hamideh Moravvej

**Affiliations:** ^1^Gastroenterology and Liver Diseases Research Center, Research Institute for Gastroenterology and Liver Diseases, Shahid Beheshti University of Medical Sciences, Tehran, Iran; ^2^Proteomics Research Center, Faculty of Paramedical Sciences, Student Research Committee, Shahid Beheshti University of Medical Sciences, Tehran, Iran; ^3^Department of Dental Science, Shahid Beheshti University of Medical Sciences, Tehran, Iran; ^4^Faculty of Medicine, Shahid Beheshti University of Medical Sciences, Tehran, Iran; ^5^Basic and Molecular Epidemiology of Gastrointestinal Disorders Research Center, Research Institute for Gastroenterology and Liver Diseases, Shahid Beheshti University of Medical Sciences, Tehran, Iran; ^6^Skin Research Center, Shahid Beheshti University of Medical Sciences, Tehran, Iran

**Keywords:** Celiac Disease, Gene, Network, Biomarker

## Abstract

**Background:**

Celiac disease (CD) is an immunological intestinal disorder, which is characterized by response to gluten. In addition to the environmental factors and dysbiosis of the gut microbiota, genetic susceptibility has an important role in the pathogenesis of this multifactorial disorder. Therefore, this study aims to present the crucial involved genes in CD pathogenesis.

**Materials and Methods:**

In this bioinformatics analysis study, significant differentially expressed genes of intraepithelial lymphocytes (IELs) samples of celiac patients versus normal patients from Gene Expression Omnibus (GEO) database were screened via the protein-protein interaction (PPI) network. The critical nodes based on degree values, betweenness centrality, and fold changes were determined and enriched by ClueGO to find relative biological terms.

**Results:**

According to the network analysis, five central nodes including IL2, PIK3CA, PRDM10, AKT1, and SRC and eight significant differentially expressed genes (DEGs) were determined as the critical genes related to CD. Also, CD4+, CD25+, alpha-beta regulatory T cell differentiation are identified as prominent biological terms in the celiac disease patients.

**Conclusion:**

There is a possible biomarker panel related to CD that can be used as a therapeutic or diagnostic tool to manage the disease.

## Introduction

Celiac disease (CD) is a small intestinal disorder that can lead to villous atrophy, malabsorption, and malignancy in the small intestine [1, 2]. CD is caused by the gluten protein in wheat, barley, and rye ingestion [3]. The main genetic predisposition factor in this disorder is the expression as HLA-DQ2 and HLA-DQ8, the antigen-presenting molecules of human leukocyte [4]. HLA-DQ2 and HLA-DQ8 bind to gluten peptides and activate destructive intestinal T cells [5]. Gluten peptides induce the secretion of IgA-class autoantibodies in the small-intestinal mucosa, which are targeted against tissue transglutaminase (tTG). Interestingly, after implementation of a gluten-free diet, these autoantibodies can disappear from the circulation more rapidly than the small-intestinal mucosal abnormality [6]. Therefore, the only approved treatment of CD is a lifelong gluten-free diet [7]. On the other hand, the histopathology of celiac disease was classified by Marsh *et al*. in 1995: Marsh I, including the increasing number of intraepithelial lymphocytes (IELs) in the mucosa of the small intestine; Marsh II, increasing the IELs and crypt hyperplasia; and Marsh III, villous atrophy [8]. T cell receptors and surface markers are used to characterize the immunological phenotype of the IELs. Study of normal duodenal biopsies revealed that IELs of healthy individuals are about 90% CD3+ and CD8+ [9]. Beside different diagnostic methods, proteomic analysis of the patient’s serum could be a clue to developing a new diagnostic and the therapeutic markers for CD [10]. Also, the protein-protein interaction network analysis obtained by proteomics assays is one of the supportive fields for discovering the pathogenesis biomarkers for celiac disease [11]. Stulík *et al*. report the detection of 11 proteins with various frequencies by sera of patients with celiac disease. They identified actin, ATP synthase b chain and two charge variants of enolase as autoantigens. Therefore, we assume that protein-protein interaction network analysis is a suitable method for screening the numerous related known genes to CD and introducing the crucial ones. The finding can be considered as potential biomarkers for prognosis and treatment.

## Material and Methods

Five gene expression profiles of intraepithelial lymphocytes samples of celiac patients were extracted from the Gene Expression Omnibus (GEO) database (GEO accession; GSE102993: link: https://www.ncbi.nlm.nih.gov/geo/query/acc.cgi?acc=GSE102993). Profiles of three clinical controls were used to be compared with the celiac samples. The cells were extracted from the small intestine (duodenal mucosa) of active celiac patients and clinical controls. The data is recorded in the database entitled “Gene expression assessed by genome-wide hybridization bead array in IELs isolated from small intestinal biopsies of celiac disease patients with active and clinical controls.” The platform is GPL6883 Illumina human ref-8 v3.0 expression beadchip. The top 250 DEGs were selected, and the uncharacterized ones were excluded. The selected significant DEGs were included in the PPI network via string database by Cytoscape software version 3.6.0 (Applied Biosystems, Foster City, CA, USA). The constructed network was analyzed by network analyzer application of Cytoscape. The hub nodes were based on degree value by using average+2SD cutoff. The 5% top nodes based on betweenness centrality were identified as bottleneck nodes [12]. The common hub and bottleneck nodes were introduced as hub-bottlenecks nodes [13]. The 8 top over-expressed and down-regulated DEGs were determined as critical DEGs. The critical DEGs and hub-bottlenecks were enriched to obtain biological terms via KEGG, wiki Pathways, REACTOME pathways, GO molecular function, GO cellular component, and GO biological process by ClueGO v2.5.0 plugin of Cytoscape. The terms were clustered based on the Kappa score. Log fold change (Log FC) >2 and P≤ 0.05 were considered as statistically significant findings.

## Results

**Figure 1:**
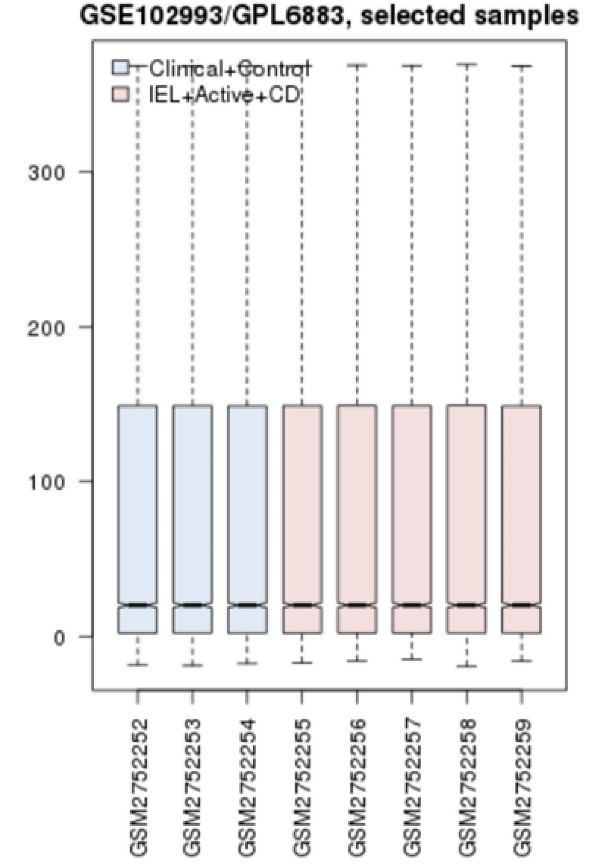
Expression profiles of 5 IELs samples of celiac patients and three controls are compared via boxplot analysis.

**Figure 2:**
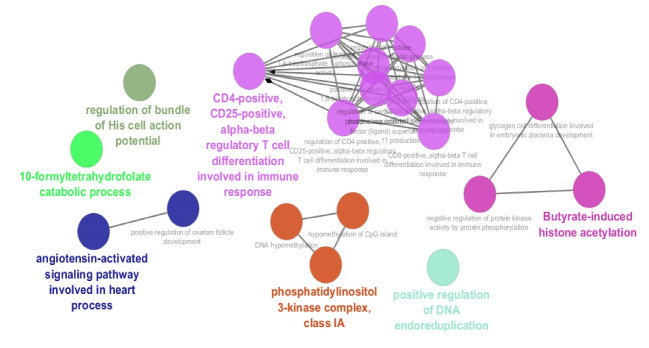
Results of gene ontology enrichment of the central nodes and the top DEGs of IELs are represented. Twenty-one biological terms via and 29 connections are clustered into seven groups. The final kappa score groups were 7, and the redundant groups with >50% overlap were merged. The largest group contains all query genes (P<0.05).

Expression profiles of 5 IELs samples of celiac patients versus controls are matched via the boxplot analysis (Figure-1). As depicted in this figure 50% of genes are expressed in higher levels relative to the other 50% genes. It is obvious that the highly expressed genes are expressed in the vast range. The samples are matched statistically and are comparable. The significant DEGs were included in a PPI network. The results indicated that a large number of genes couldn’t interact with each other, so 100 relevant and additional genes were added to the query genes. The resulting network including 60 isolated nodes, one double nodes component, and a main connected component was constructed. The main connected component contains 257 nodes and 4741 edges. The network was characterized as a scale-free network, and most of the isolated nodes were included in the network. The main connected component was analyzed to identify the central nodes. The hub-bottleneck nodes of the PPI network of IELs in celiac patients in comparison with the healthy control samples including IL2, PIK3CA, PRDM10, AKT1, and SRC are shown in Table-1. Since expression physicochemical properties of the genes are the two important features of the nodes, the expression parameter was considered. The selected top up-regulated and down-regulated DEGs of IELs in celiac patients are determined and shown in Table-2. Since gene function plays an important role in the molecular mechanism of diseases, gene ontology of the identified central nodes and the top DEGs was done, and the biological processes, cellular component, molecular function, and biochemical pathways related to these critical nodes were determined (Figure-2).

## Discussion

Molecular mechanisms of different diseases are studied via PPI network analysis. These findings led to the introduction of several central genes, which potentially can be considered as a biomarker panel. In this study, IELs are targeted to achieve new aspects of the molecular mechanism of celiac disease. As it is shown in Figure-1, the samples are comparable because the expression profiles are matched via boxplot analysis. Among numerous DEGs, only the limited numbers of genes were included in the PPI network. As it is represented in Table-1, there are five central nodes that play a crucial role in the network. The central nodes and also the deregulated (Table-2) are suitable genes as a biomarker panel. In the following part, the roles of these critical genes in celiac disease will be described and discussed: IL2 the top hub-bottleneck node is a highly connected node that interacts with 113 nodes directly [14]. IL2, as an important lymphokine, is involved in several cellular processes of T cells. The cells which secret IL2 are responsible for responses to antigenic or mitogenic stimulation [15]. Nilsen *et al*. (1998) reported that levels of interferon-γ, IL2, IL4, IL6, and tumor necrosis factor-α in the duodenal biopsy of the treated celiac patients which were exposed to gluten were rapidly elicited [16]. The regulation relationship between SRC and AKT isoforms, which play a critical role in cell survival, growth, proliferation, angiogenesis, metabolism, and migration is emphasized. These two genes originally are known as oncogenes [17]. The pivotal role of PRDM proteins in cell growth, differentiation, and also neoplastic transformation is highlighted [18]. Therefore, SRC, AKT, and PRDM are involved in regulation of cell differentiation and growth, and deregulation of these terms is the important processes in cancer. ADGRE1 is introduced as a macrophage marker, and its role in several phenomena such as bone regeneration is highlighted [19]. ALDH1L2 is a member of ALDH superfamily, which plays key roles in various life processes mostly in detoxification of pharmaceuticals and environmental pollutants. The process proceeds via NAD (P)+-dependent oxidation aldehyde substrates [20]. As it is shown in Table-2, ADGRE1 and ALDH1L2 are the top up-regulated and down-regulated DEGs. As depicted in Figure-2, CD4-positive, CD25-positive, alpha-beta regulatory T cell differentiation involved in the immune response is the prominent biological processes related to the critical genes. All query genes including DEGs and central nodes are involved in this cluster. Based on our finding the T cells are essential elements in celiac diseases. Intestinal T cell response in celiac diseases is investigated and discussed in several types of research [21, 22]. As Jabri and Sollid reported, the main feature of the molecular mechanism of celiac disease is related to the response of CD4 T cells to dietary gluten. The response that promotes antigen-antibody reactions results in the most developed lesion in the proximal small intestine of patients [23]. Gene ontology finding reflects direct involvement of the important genes in CD. The 13 introduced critical genes may be a suitable biomarker panel or at least numbers of them can be considered as individual biomarkers of CD; however, more investigation is needed to achieve these successes.

## Conclusion

In conclusion, achieving a molecular diagnostic tool for CD is feasible. The introduced possible biomarkers are suitable therapeutic reagents if further investigation is planned.

## Acknowledgment

This project was supported by Shahid Beheshti University of Medical Sciences (Grant Nubmer 15369).

## Conflict of Interest

There is no conflict of interest.

**Table 1:** The Hub-Bottleneck Nodes of the PPI Network of IELs in Celiac Patients

**R**	**Name**	**Description**	**BC**	**D**
**1**	IL2	interleukin 2	0.024	113
**2**	PIK3CA	phosphatidylinositol-4,5-bisphosphate 3-kinase, catalytic subunit alpha	0.019	112
**3**	PRDM10	PR domain containing 10	0.031	109
**4**	AKT1	v-akt murine thymoma viral oncogene homolog 1	0.032	108
**5**	SRC	v-src sarcoma (Schmidt-Ruppin A-2) viral oncogene homolog (avian)	0.024	107

**BC:** Betweenness centrality; **D:** Degree

**Table 2:** The Top 4 Up-regulated and 4 Down-regulated DEGs of IELs in Celiac Patients

**R**	**Log FC**	**Name**	**Description**
**1**	7.21	ADGRE1	adhesion G protein-coupled receptor E1
**2**	6.48	RANGRF	RAN guanine nucleotide release factor
**3**	6.22	IFNG	interferon gamma
**4**	6.13	PARP15	poly(ADP-ribose) polymerase family member 15
**5**	-4.03	UNC93A	unc-93 homolog A (C. elegans)
**6**	-4.04	INS-IGF2	INS-IGF2 read-through
**7**	-4.47	SLC22A7	solute carrier family 22 member 7
**8**	-5.22	ALDH1L2	aldehyde dehydrogenase 1 family member L2

**FC:** Fold change
